# Assessment of abdominal obesity prevalence and determinants among adults in southwest Ethiopia: a cross-sectional study

**DOI:** 10.3389/fpubh.2024.1400066

**Published:** 2024-10-18

**Authors:** Tewodros Yosef, Asresash Sebeta, Eyob Tekalign, Binyam Girma Sisay, Bayu Begashaw Bekele, Aragaw Tesfaw, Nigusie Shifera

**Affiliations:** ^1^School of Public Health, College of Medicine and Health Sciences, Mizan-Tepi University, Mizan Teferi, Ethiopia; ^2^School of Medicine, Faculty of Health, Deakin University, Waurn Ponds, VIC, Australia; ^3^Department of Emergency Medicine, Mizan-Aman College of Health Sciences, Mizan Teferi, Ethiopia; ^4^Department of Medical Laboratory Science, College of Medicine and Health Sciences, Mizan-Tepi University, Mizan Teferi, Ethiopia; ^5^School of Exercise and Nutrition Sciences, Institute for Physical Activity and Nutrition, Deakin University, Melbourne, VIC, Australia; ^6^Division of Public Health Sciences, Department of Surgery, Washington University School of Medicine, St Louis, MO, United States; ^7^School of Public Health, College of Health Sciences, Debre Tabor University, Debre Tabor, Ethiopia

**Keywords:** abdominal obesity, cross-sectional study, prevalence ratio, southwest Ethiopia, waist-to-hip ratio

## Abstract

**Background:**

Abdominal obesity, excess fat around the abdomen, is more harmful than other fat types and is a key diagnostic criterion for metabolic syndrome. It poses a growing global public health concern. This study aimed to assess the prevalence of and determinants of abdominal obesity among adults in southwest Ethiopia.

**Methods:**

A cross-sectional study involving 624 adults in Semen Bench district, southwest Ethiopia was conducted from May 23 to June 23, 2022. The data was gathered using questionnaires and anthropometric measurements. The data were analyzed using Stata version 18. A robust Poisson regression was used due to the abdominal obesity prevalence exceeding 10%. Variables with *p*-values < 0.25 in the bivariate analysis were included in the multivariable analysis. The adjusted prevalence ratio (APR) and 95% confidence interval were reported to indicate statistical significance and the strength of associations.

**Results:**

The prevalence of abdominal obesity was 18% (95% CI: 15–21%). After adjusting for confounding variables, the determinants of abdominal obesity included participants aged 31–50 (APR = 3.62, 95% CI: 2.67–8.95) and 51–65 (APR = 3.24, 95% CI: 2.51–8.69), being female (APR = 2.65, 95% CI: 1.63–4.98), having a low wealth index (APR = 1.46, 95% CI: 1.19–3.76), physical inactivity (APR = 1.39, 95% CI: 1.06–4.18), lack of adequate dietary diversity (APR = 3.08, 95% CI: 2.09–6.42), and food insecurity (APR = 2.84, 95% CI: 1.82–7.68).

**Conclusion:**

The research revealed that 18% of the participants exhibited abdominal obesity. Factors such as advanced age, being female, having a low wealth index, physical inactivity, insufficient dietary diversity, and food insecurity were identified as contributors to abdominal obesity. Therefore, it is crucial to develop targeted interventions to address modifiable factors, as this can also help prevent the onset of non-communicable chronic diseases associated with abdominal obesity.

## Introduction

The World Health Organization (WHO) states that obesity is one of the most prevalent public health issues. Yet, it remains one of the most overlooked, affecting both developed and developing countries (1). Obesity is the excessive fat buildup that can impair overall health (2). Obesity has become a major global health problem, with its prevalence more than doubling since 1980 (1). Obesity, once considered an issue exclusive to high-income countries, has rapidly increased in low- and middle-income nations over the past 30 years (3). In low and middle-income countries, a significant portion of the adult population, particularly in urban areas, is affected by overweight and obesity (4, 5).

Obesity can be classified as general or abdominal. General obesity is defined by body mass index (BMI). Abdominal obesity is determined by waist circumference (WC) or waist-to-hip ratio (WHR) (6). Abdominal obesity, characterized by the accumulation of fat in the abdominal area, has risen markedly in recent years, leading to increased health risks (7). This type of fat is particularly detrimental compared to others and serves as a crucial indicator in diagnosing metabolic syndrome (8). Abdominal obesity is known to harm health, increasing the risk of diabetes, hypertension, dyslipidemia, cardiovascular disease, stroke, and cancer (9). Abdominal obesity is notably associated with increased mortality in all weight categories (10).

In Sub-Saharan Africa, abdominal obesity is increasing, with higher rates in urban areas compared to rural ones. It is often viewed as a sign of affluence and respect (11). Multiple studies in West Africa have reported abdominal obesity prevalence rates ranging from 22.5 to 50.8% (12–14). Several studies in East Africa have revealed that the prevalence of abdominal obesity ranges from 11.8 to 67.8% (15–19).

Overeating energy-dense foods increases the risk of abdominal obesity, common in sedentary modern lifestyles (20). Various factors contributing to abdominal obesity include sociodemographic aspects like age (13, 17, 21–24), gender (21, 25), wealth index (21, 23–25), marital (22–24), and educational status (15, 23), and place of residence (15, 22, 26). Behavioral factors such as sedentary lifestyles (22, 26), alcohol use and smoking habits (26, 27), fast food consumption (24), reduced physical activity (21, 25, 26), dietary diversity (23, 25), low intake of fruits and vegetables, and higher consumption of processed foods (28), and snacking habits (23) also play significant roles. Additionally, comorbidities such as hypertension (13, 15, 21), overweight, obesity (13, 21), and knowledge about obesity (24) contribute to this condition across different studies.

Ethiopia is experiencing a significant rise in obesity rates (29). Abdominal obesity, specifically, is becoming more common across the country, with reported prevalence rates ranging from 16.5 to 28.4% (23–25). However, specific evidence regarding this issue in the study area is currently lacking. Thus, this study aimed to determine the prevalence of abdominal obesity and explore its determinants among adults in southwest Ethiopia.

## Methods

### Study setting, design, and period

A community-based cross-sectional survey was conducted among adults residing in the Semen Bench district, Bench Sheko zone in southwest Ethiopia from May 23 to June 23, 2022. Semen Bench district is one of the 10 decentralized districts in the Bench Sheko zone. It is located 17 km from the zonal administration town of Mizan-Aman and 568 km southwest of Ethiopia’s capital, Addis Ababa. The residents primarily rely on crop cultivation and livestock breeding for their livelihoods.

### Populations

The source population included all adults aged ≥18 years in the Semen Bench district, while the study population consisted of randomly selected adults who had lived there for at least 6 months during data collection. Exclusions included women who gave birth in the last 6 months, and adults with abdominal distension, severe illness, communication barriers, or physical deformities affecting measurement.

### Sample size determination and sampling technique

The sample size was calculated using the formula for a single population proportion, assuming a 95% confidence level and a 5% margin of error, with a prevalence of abdominal obesity at 24.4% (25). Adjustments were made for a 10% non-response rate and a design effect of 2. The final sample size was 624. A stratified sampling technique was used, dividing the district into urban, semi-urban, and rural kebeles. One-third of kebeles from urban and semi-urban were randomly selected. The total households in each kebele were divided by the allocated sample sizes to determine sampling intervals (k). A random start was chosen by lottery, and every kth household from the random start was included until the required sample size was reached.

### Data collection tools, and procedures

The study used a modified questionnaire adapted from the WHO-STEP approach for chronic non-communicable diseases (30). All participants underwent interviews, and multiple anthropometric measurements were conducted, with recorded averages. Waist and hip circumference were measured to the nearest 0.1 cm using a tape measuring, and the waist-to-hip ratio was then calculated. Principal component analysis was used to compute the household wealth index. The wealth index was divided into higher, medium, and lower categories (31). The study used the Household Food Insecurity Access Scale (HFIAS) to assess household food insecurity. Participants answered nine questions about their household’s food experiences in the past 30 days, focusing on worry about food access (1 question), food quality (3 questions), and inadequate food intake and its effects (5 questions). Responses were scored from 0 to 3 based on frequency: 0 for never, 1 for rarely (once or twice), 2 for sometimes (three to 10 times), and 3 for often (more than 10 times). A score of 27 indicates severe food insecurity, while lower scores indicate greater food security (32). The Food Frequency Questionnaire (FFQ) assessed participants’ dietary habits across eight categories: meat, eggs, fish, fat-rich foods, vegetables, fruits, dairy products, and sweets. Consuming four or more food categories within 24 h in households indicates sufficient dietary diversity, whereas consuming fewer than four food types within the same timeframe suggests insufficient diversity (33). A pretest involving 5% of the sample was conducted in Mizan Aman town before data collection. Additionally, comprehensive training was provided to data collectors and supervisors to ensure the questionnaire was administered consistently and clearly.

### Study variables

The study considered abdominal obesity as the dependent variable. Independent variables included sociodemographic factors (age, sex, marital status, education, occupation, family size, wealth index, and place of residence), behavioral characteristics (alcohol consumption, cigarette smoking, khat chewing, and physical activity), and nutritional factors (dietary diversity, household food security, dining out of home, skipping breakfast, snacking habits, fruit and vegetable intake).

### Operational definitions

A waist-to-hip ratio above 0.85 for women and 0.90 for men indicates abdominal obesity (34). Alcohol drinkers: those who drink alcohol (beer, local beer or areke, tella, or tej) daily or every other day (35, 36). Cigarette smokers: those who smoke daily, regardless of the number (35–37).

Khat (*Catha edulis*) is a stimulant derived from the fresh leaves of the *Catha edulis* shrub, native to East Africa and southern Arabia. Khat chewers: those who chew khat at least once per week (35, 36). Physically active: participants with 3+ days of physical activity (walking, running, bicycling, and stretching exercises such as sit-ups and pull-ups) (30 min/day) per week (36). A snack was characterized as consuming extra food items such as dabo kolo (small pieces of baked bread resembling pretzels), kolo (roasted barley, sometimes mixed with other local grains), sambusas (fried or baked pastries filled with lentils, meat, or vegetables), as well as bread and fruits (such as mangoes, papayas, and bananas), between the three main meals of the day, regardless of quantity (38).

### Statistical analysis

The data were analyzed using Stata version 18 (Stata Corp. 2023. Stata Statistical Software: Release 18. College Station, TX: Stata Corp LLC). Continuous variables were assessed for normality, normally distributed variables were summarized using means and standard deviations (SD), while non-normally distributed variables were summarized using medians and interquartile ranges (IQR). Categorical variables were summarized using frequencies and percentages. This research utilized a cross-sectional design and identified that the prevalence of abdominal obesity was over 10%. Given that the odds ratio could overstate the relationship between abdominal obesity and the independent variables in such instances, the prevalence ratio was deemed a more accurate measure of association. To determine the predictors of abdominal obesity, a Poisson regression with robust variance was applied. Variables with a *p-*value < 0.25 in the initial bivariate analysis were included in the multivariable model. The model’s fit was evaluated using deviance, and the model with the lowest deviance was selected as the best fit. The study reported the Adjusted Prevalence Ratio (APR) along with its 95% confidence interval (CI), and considered variables with a *p*-value < 0.05 in the multivariable analysis as significant predictors of abdominal obesity.

## Results

### Sociodemographic and economic characteristics

All respondents participated in the study, achieving a 100% response rate. Of 624 participants, 47.4% were female. The average age of participants was 33.5 (SD ± 7.9) years, with half falling between 20 and 30 years old. The median family size was 3 (IQR 1–7). Additionally, 18.3% of participants lived in households with five or more members. Out of the participants, 383 (61.4%) had formal education, and 255 (40.9%) were categorized as having a low wealth index (Table 1).

**Table 1:** Sociodemographic characteristics of participants in southwest Ethiopia.

Variables	Categories	Frequency (*n*)	Percent (%)
Sex	Male	328	52.6
	Female	296	47.4
Age group [33.5 ± 7.9] years	20–30	311	49.8
	31–40	205	32.9
	41–50	65	10.4
	51–65	43	6.9
Education	Not formally educated	241	38.6
	Formally educated	383	61.4
Occupation	Merchant	238	38.1
	Government employee	167	26.8
	Housewives	100	16.0
	Farmer	65	10.4
	Daily laborer	54	8.7
Marital status	Married	386	61.9
	Not married	238	38.1
Family size [3 (1–7)]	<5	510	81.7
	≥5	114	18.3
Wealth index	Low	255	40.9
	Medium	156	25.0
	High	213	34.1
Weight (kg)	78.4 ± 11.2		
Height (m)	1.76 ± 0.11		
Body mass index (kg/m^2^)	28.4 ± 1.73		

Data are presented as mean (±SD) and median (IQR).

### Behavioral, nutrition-related characteristics and prevalence of abdominal obesity

**Figure 1:**
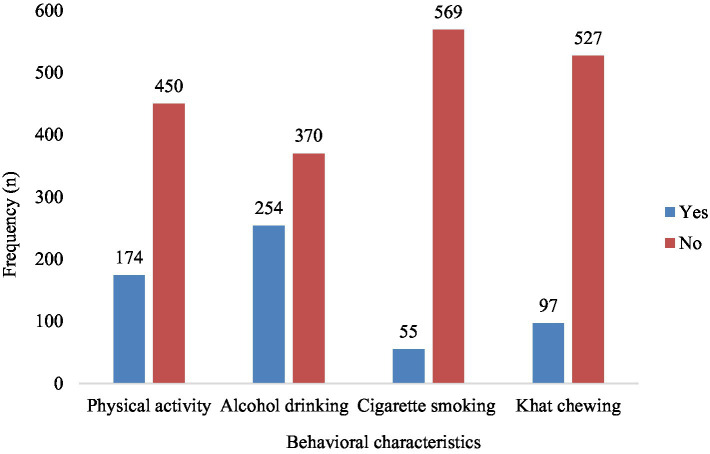
Behavioral characetristics of the participants in southwest Ethiopia.

**Figure 2:**
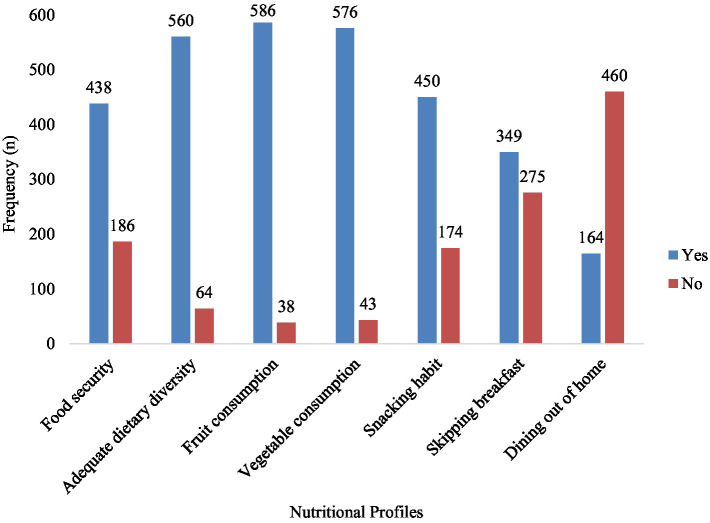
Nutritional profiles of participants in southwest Ethiopia.

**Figure 3:**
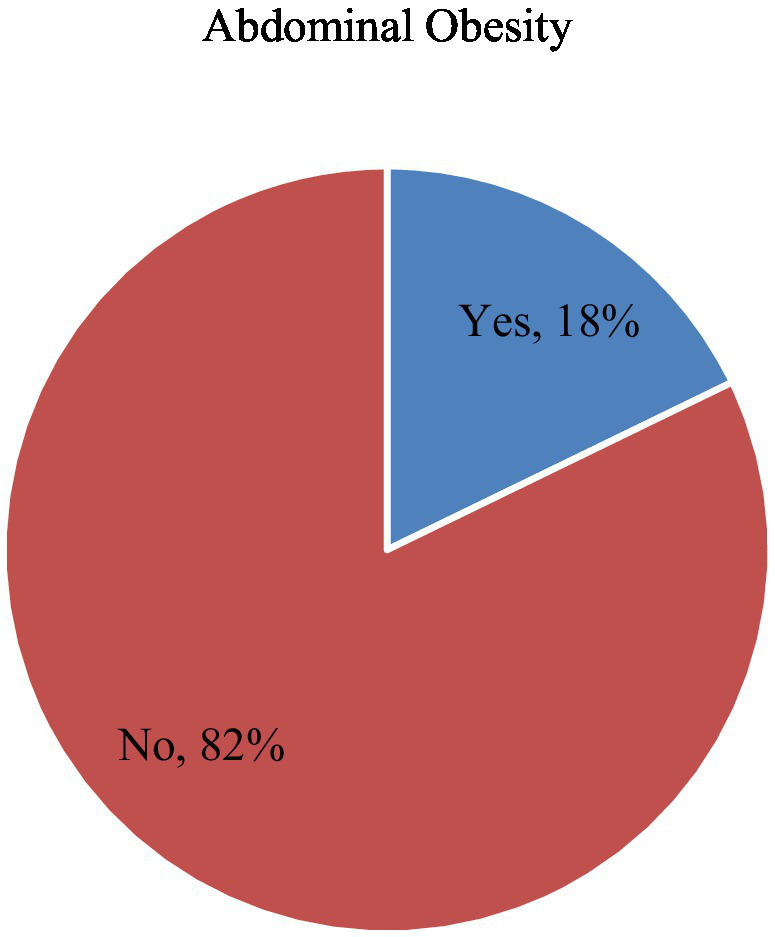
The prevalence of abdominal obesity among participants in southwest Ethiopia.

Among participants, 40.7% reported drinking alcohol, 8.8% smoked cigarettes, 15.6% chewed khat, and 27.9% engaged in physical activity (Figure 1). In the study, 70.2% of the participants were from food-secure households. Additionally, 72.1% had a snacking habit, 63.1% skipped breakfast, and the consumption rates for fruit, and vegetables were 93.9, and 92.3%, respectively (Figure 2). The prevalence of abdominal obesity was 18% (95% CI: 15–21%) (Figure 3).

### Factors associated with abdominal obesity

After adjusting for confounding variables, the determinants of abdominal obesity included participants aged 31–50 (APR = 3.62, 95% CI: 2.67–8.95) and 51–65 (APR = 3.24, 95% CI: 2.51–8.69), being female (APR = 2.65, 95% CI: 1.63–4.98), having a low wealth index (APR = 1.46, 95% CI: 1.19–3.76), physical inactivity (APR = 1.39, 95% CI: 1.06–4.18), lack of adequate dietary diversity (APR = 3.08, 95% CI: 2.09–6.42), and food insecurity (APR = 2.84, 95% CI: 1.82–7.68) (Table 2).

**Table 2:** Factors associated with abdominal obesity among participants in southwest Ethiopia.

Variables	Categories	Abdominal obesity		CPR, 95%CI	APR, 95%CI	*p*-value
		Yes	No			
Age	20–30	39	272	1	1	
	31–40	21	184	0.82 (0.68, 3.24)*	0.75 (0.61, 2.89)	0.063
	41–50	32	33	3.93 (2.53, 9.61)**	3.62 (2.67, 8.95)	**0.009**
	51–65	20	23	3.71 (2.49, 9.52)**	3.24 (2.51, 8.69)	**0.032**
Sex	Male	34	294	1	1	
	Female	78	218	2.54 (1.58, 4.92)**	2.65 (1.63, 4.98)	**<0.001**
Marital status	Married	64	322	1	1	
	Not married	28	210	0.71 (0.54, 2.83)*	0.67 (0.61, 2.28)	0.283
Educational status	Not formally educated	52	189	1.38 (0.78, 3.89)*	1.35 (0.70, 4.67)	0.187
	Formally educated	60	323	1	1	
Wealth index	Low	52	203	1.50 (1.32, 4.29)**	1.46 (1.19, 3.76)	**0.028**
	Medium	31	125	1.46 (0.79, 3.26)*	1.41 (0.72, 3.21)	0.281
	High	29	184	1	1	
Physical activity	Yes	24	150	1	1	
	No	88	362	1.42 (1.11, 4.49)**	1.39 (1.06, 4.18)	**0.004**
Alcohol consumption	Yes	66	188	2.09 (0.82, 4.56)*	2.03 (0.76, 4.78)	0.534
	No	46	324	1	1	
Adequate dietary diversity	Yes	82	478	1	1	
	No	30	34	3.20 (1.89, 6.53)**	3.08 (2.09, 6.42)	**<0.001**
Food security	Yes	50	388	1	1	
	No	62	124	2.92 (1.96, 7.68)**	2.84 (1.82, 7.68)	**<0.001**
Skipping breakfast	Yes	59	290	0.87 (0.65, 2.14)*	0.82 (0.60, 2.08)	0.223
	No	53	222	1	1	

1 = reference value; *p-value < 0.25; **p-value < 0.05; CPR, crude prevalence ratio; APR, adjusted prevalence ratio; CI, confidence interval. Bold shows statistical significance at a *p*-value < 0.05.

## Discussion

This study sought to assess the prevalence of abdominal obesity and its determinants among adults in the Semen Bench district, in southwest Ethiopia. The prevalence of abdominal obesity was 18% (95% CI: 15–21%). Factors associated with abdominal obesity included age, sex, wealth index, physical activity, dietary diversity, and food security.

The prevalence of abdominal obesity (18%) in this study aligned with findings from Woldia, Ethiopia (16.5%) (23). It was higher than rates reported in Uganda (11.8%) (15) but lower than those in Dilla (24.4%) (25), Nekemte (28.4%) (24), and Bale Zone (39.0%) (39) in Ethiopia, as well as Abia State, Nigeria (21.8%) (40), and Tanzania (24.9%) (22). Variations in abdominal obesity prevalence across studies stem from differences in sampled populations, methodological discrepancies like sample size and measurement techniques (24, 39), geographical and cultural influences on diet and physical activity, disparities in healthcare access and economic development, and evolving lifestyle behaviors and healthcare interventions targeting obesity.

The age of participants is significantly associated with abdominal obesity. Participants aged 41–50 and 51–65 years had increased prevalence of abdominal obesity compared to those aged 20–30 years. This finding was supported by other studies somewhere (13, 17, 21–24). This could be explained by the fact that advanced age is associated with abdominal obesity due to slower metabolism, muscle loss, hormonal changes, reduced physical activity, and fat redistribution to the abdominal region.

This study revealed a significant association between sex and abdominal obesity. Female participants had a higher prevalence of abdominal obesity compared to males, a finding supported by other studies elsewhere (21, 23, 25, 27, 41). This can be explained by the fact that females tend to develop abdominal obesity due to higher estrogen levels, sedentary lifestyles influenced by cultural roles, effects of pregnancy and childbirth, dietary habits that increase fat intake, and genetic predispositions to store abdominal fat (42). Traditional gender roles in Ethiopian culture often lead women to engage in less physically demanding activities than men.

Individuals with a low wealth index had a higher prevalence of abdominal obesity compared to those with a high wealth index, a finding supported by other studies (21, 23–25). Low wealth can lead to the consumption of inexpensive, unhealthy foods, increasing the risk of abdominal obesity. Lee et al. (43) found that poor-quality diets lacking nutrients may also contribute to abdominal fat accumulation.

Physical activity is significantly associated with abdominal obesity. Physically inactive participants were more likely to be abdominally obese compared to physically active participants. This finding was consistent with other studies elsewhere (21, 25, 26). Physical inactivity leads to abdominal obesity by reducing energy expenditure, metabolism, and insulin sensitivity while disrupting hormonal balance, promoting muscle loss, and increasing the risk of metabolic and cardiovascular diseases linked to abdominal fat accumulation (44). Exercise helps fight belly fat by burning calories and boosting metabolism.

Participants who were food-insecure had a higher prevalence of abdominal obesity compared to their counterparts. This finding was supported by other studies (45, 46). Food insecurity is linked to abdominal obesity due to irregular eating patterns and reliance on cheap, calorie-dense, nutrient-poor foods, leading to weight gain and abdominal fat (43, 47). Stress and anxiety from food insecurity can also cause overeating, further increasing obesity risk.

Participants with no dietary diversity were more likely to be abdominally obese compared to their counterparts. This finding was consistent with other studies (23, 25). A lack of dietary diversity is linked to abdominal obesity due to reliance on high-calorie, low-nutrient foods, leading to excess calorie intake and poor nutrition (43, 47). A diverse diet, rich in fruits, vegetables, whole grains, and lean proteins, helps maintain a healthy weight and prevent obesity.

Over 90% of participants in the area consumed fruits and vegetables, a higher rate than 67.87% in Jimma town (38). This difference may be attributed to the area’s abundant fruit and vegetable production, supported by year-round rainfall. Eating fruits and vegetables can reduce abdominal obesity by providing low-calorie, high-fiber, and nutrient-dense options that promote fullness and digestion. They also support overall health, improve metabolism and insulin sensitivity, and help decrease the intake of high-calorie, processed foods (48). Additionally, their high water content aids in hydration and satiety. Given these benefits, health education campaigns should be developed to raise community awareness about the importance of consuming fruits and vegetables to reduce the incidence of abdominal obesity.

### Strength and limitations

This study stands out for its novelty as the first of its kind in the area, offering valuable insights and baseline data for informing future research and public health interventions. Additional strengths include using a modified standard tool, achieving a 100% response rate, and employing a random sampling method. However, its focus on a single area limits the generalization of the findings to other regions or populations. Additionally, the cross-sectionality of the study presents a limitation, as it captures data at a single point in time, making it difficult to establish causal relationships or temporal links between variables. Relying on self-reported data for physical activity, dietary diversity, and food insecurity may introduce recall bias and affect accuracy in assessing associations with abdominal obesity. Longitudinal studies would be needed to understand the dynamics and causation of the observed factors.

## Conclusion

The study found that 18% of participants had abdominal obesity, influenced by factors such as advanced age, female gender, low wealth index, physical inactivity, inadequate dietary diversity, and food insecurity. Addressing the modifiable factors through targeted interventions is crucial to preventing non-communicable chronic diseases associated with abdominal obesity, including cardiovascular disorders, type 2 diabetes, and certain cancers. Promoting physical activity, improving dietary habits, and addressing socioeconomic disparities can significantly reduce the burden of abdominal obesity and enhance overall public health outcomes.

## Data availability statement

The raw data supporting the conclusions of this article will be made available by the authors, without undue reservation.

## Ethics statement

The studies involving humans were approved by Mizan-Tepi University College of Medicine and Health Sciences research and community service review committee. The studies were conducted in accordance with the local legislation and institutional requirements. The participants provided their written informed consent to participate in this study.

## Author contributions

TY: Conceptualization, Data curation, Formal analysis, Funding acquisition, Investigation, Methodology, Project administration, Resources, Software, Supervision, Validation, Visualization, Writing – original draft, Writing – review and editing. AS: Formal analysis, Funding acquisition, Investigation, Project administration, Resources, Supervision, Writing – original draft, Writing – review and editing. ET: Formal analysis, Funding acquisition, Investigation, Project administration, Resources, Supervision, Writing – original draft, Writing – review and editing. BS: Formal analysis, Funding acquisition, Investigation, Project administration, Resources, Supervision, Writing – original draft, Writing – review and editing. BB: Formal analysis, Funding acquisition, Investigation, Project administration, Resources, Supervision, Writing – original draft, Writing – review and editing. AT: Formal analysis, Investigation, Project administration, Software, Supervision, Visualization, Writing – original draft, Writing – review and editing. NS: Formal analysis, Investigation, Methodology, Software, Validation, Visualization, Writing – original draft, Writing – review and editing.
